# Inhibition of AKT survival pathway by a small molecule inhibitor in human endometrial cancer cells

**DOI:** 10.1038/sj.bjc.6602214

**Published:** 2004-10-26

**Authors:** X Jin, D R Gossett, S Wang, D Yang, Y Cao, J Chen, R Guo, R K Reynolds, J Lin

**Affiliations:** 1 Department of Pathology, University of Michigan Comprehensive Cancer Center, Ann Arbor, MI, USA; 2 Department of Obstetrics and Gynecology, University of Michigan Comprehensive Cancer Center, Ann Arbor, MI, USA; 3 Division of Hematology and Oncology, Department of Internal Medicine, University of Michigan Comprehensive Cancer Center, Ann Arbor, MI, USA; 4 Center for Childhood Cancer, Columbus Children's Research Institute, The Ohio State University, Columbus, OH 43205, USA

**Keywords:** AKT, PTEN, endometrial cancer, apoptosis, experimental therapeutics

## Abstract

The PTEN (phosphatase and tensin homolog deleted on chromosome 10) tumour suppressor is mutated in 40–50% of human endometrial cancers. PTEN exerts its effects in part via inhibition of the antiapoptotic protein AKT. We demonstrate that two endometrial cancer cell lines that harbour *PTEN* mutations, Ishikawa and RL95-2, have high levels of phosphorylated AKT and high AKT kinase activity. Two additional endometrial cancer cell lines that express wild-type *PTEN*, Hec1A and KLE, have little phosphorylated AKT and minimal demonstrable AKT kinase activity. We tested a potential inhibitor of the AKT pathway, API-59CJ-OMe, in these four cell lines. We found that API-59CJ-OMe inhibits AKT kinase activity and induces apoptosis in the Ishikawa and RL95-2 cell lines with high AKT activity, but has little effect on Hec1A and KLE cells without AKT activity. API-59CJ-OMe may therefore have therapeutic potential for those endometrial cancers that harbour PTEN mutations and AKT activation.

Endometrial cancer is the most common gynaecologic malignancy in developed countries, with approximately 40 000 new diagnoses each year in the US alone. Mutations of the tumour suppressor PTEN are found in 40–50% of human endometrial cancers (Tashiro *et al*, 1997; Ali, 2000). PTEN is also frequently mutated in brain, breast, and prostate cancers (Cairns *et al*, 1997; Li and Sun, 1998; Davies *et al*, 1999). PTEN is thought to function as a tumour suppressor due to its ability to block G_1_ cell cycle progression, induce apoptosis, and negatively regulate the PI3-K/AKT cell survival pathway (Stambolic *et al*, 1998). Accordingly, in cancer cells with PTEN mutation or deletion, AKT activity is dramatically elevated (Kanamori *et al*, 2001). AKT is a serine/threonine kinase that is activated in response to growth factors or cytokines by a mechanism involving phosphoinositide 3-kinase (PI3-K) and phosphoinositide-dependent kinase-1 (PDK-1) (Franke *et al*, 1997; Kulik *et al*, 1997). AKT has three isoforms: AKT1 (PKB*α*), AKT2 (PKB*β*), and AKT3 (PKB*γ*) (Datta *et al*, 1999). These isoforms have greater than 85% sequence identity and have the same structural organisation. Amplification of the *AKT2* oncogene and high AKT kinase activity have been detected in breast, pancreatic, ovarian, brain, prostate, and gastric cancers (Cheng *et al*, 1992; Bellacosa *et al*, 1995).

AKT provides a survival signal that protects cells from apoptosis induced by various stresses (Franke *et al*, 1997; Kulik *et al*, 1997). Some examples of the known mechanisms by which AKT prevents apoptosis are the phosphorylation of Bad, glycogen synthase kinase-3 (GSK-3), forkhead transcription factor (FKHR), and caspase-9 (Del Peso *et al*, 1997; Cardone *et al*, 1998; Pap and Cooper, 1998; Brunet *et al*, 1999). Phosphorylation of these proteins results in inactivation of their apoptotic functions. AKT may suppress apoptosis by stimulating the transactivation potential of the RelA/p65 subunit of NF-kappaB (Romashkova and Makarov, 1999; Madrid *et al*, 2000). AKT may also enhance the ubiquitination-promoting function of Mdm2, resulting in ubiquitin-mediated degradation of the tumour suppressor p53 (Ogawara *et al*, 2002).

Development of potent AKT inhibitors is a promising therapeutic strategy for endometrial carcinoma. We have employed a bioinformatics approach to identify potential inhibitors of the AKT pathway (Wang and Yang, manuscript in preparation). We first performed a Western blot analysis to probe the level of phosphorylated AKT (p-AKT) in the National Cancer Institute (NCI) 60 human cancer cell lines. Correlation analysis was performed of the *in vitro* anticancer activity of 35 000 compounds in the NCI's anticancer database, and the p-AKT levels in the NCI 60 human cancer cell lines to identify compounds whose *in vitro* anticancer activities significantly correlated with the p-AKT level in the 60 cancer cell lines. Compounds whose *in vitro* anticancer activities significantly correlated with the p-AKT level in the 60 cancer cell lines were considered as candidate inhibitors for the AKT pathway. API-59CJ-OMe (9-methoxy-2-methylellipticinium acetate) was identified as a potential inhibitor. Our further evaluations in human prostate and breast cancer cell lines showed that API-59CJ-OMe potently inhibits cell growth and induces apoptosis in cell lines with high levels of p-AKT, but has minimal activity in cell lines with low levels of p-AKT (Wang and Yang, manuscript in preparation), suggesting that API-59CJ-OMe may target the AKT pathway.

In the present study, we tested API-59CJ-OMe in PTEN-defective endometrial cancer cells. We found that API-59CJ-OMe selectively inhibits AKT kinase activity and induces apoptosis in endometrial cancer cell lines expressing high levels of AKT activity. API-59CJ-OMe has little effect in endometrial cancer cells lacking AKT activity. This is the first report of a potential AKT inhibitor in endometrial cancer.

## MATERIALS AND METHODS

### Cell lines

Hec1A, RL95-2, and KLE human endometrial cancer cell lines were purchased from American Type Culture Collection (Manassas, VA, USA). Ishikawa human endometrial cancer cell line has been previously described and was obtained from Dr Masato Nishida (Holinka *et al*, 1986). Cells were maintained in 90% Dulbecco's modified Eagle's medium supplemented with 10% fetal bovine serum and antibiotics (5000 U ml^−1^ penicillin G, 5000 *μ*g ml^−1^ streptomycin) (Gibco/BRL Life Technologies, Inc., Rockville, MD, USA) at 37°C in 5% CO_2_.

### Western blot analysis

To analyse levels of phosphorylation of AKT, PDK1, and ERK1/2 proteins, cells were plated at 1.2 × 10^6^ cells. 100 mm^−1^ dish 1 day prior to treatment. Cells were then treated with API-59CJ-OMe at the indicated dose for 48 or 72 h before harvesting. In total, 100 *μ*g of total protein from cell lysates was separated on 10% SDS–polyacrylamide gels and blotted with a 1 : 1000 dilution of antibodies against phospho-AKT (Ser 473 or Thr 308), phospho-PDK1 (Ser 241), phospho-ERK1/2 (Thr202/Tyr204), phospho-JNK (Thr 183/Tyr 185), phospho-SGK (Ser 78), phospho-PKC*ζ*/*λ* (Thr 410/403), phospho-PKC*α*/*β* (Thr 638/641), total AKT, or total PTEN (Cell Signaling Tech., Beverly, MA, USA). The same membranes were analysed with a 1 : 2500 dilution of anti-GAPDH (glyceraldehyde-3-phosphate dehydrogenase) monoclonal antibody (Chemicon International, Inc., Temecula, CA, USA) as a protein loading control. All blots were incubated with 1 : 10 000 dilution of secondary alkaline phosphatase-conjugated anti-mouse or anti-rabbit antibody (Amersham Pharmacia Biotech., Piscataway, NJ, USA). Blots were scanned with ImageQuant Software using an ECF Western blotting detection system (Amersham Pharmacia Biotech., Piscataway, NJ, USA) on a Molecular Dynamics STORM PhosphorImager (Sunnyvale, CA, USA). Each Western blot was performed a minimum of three times.

### AKT and ERK kinase assays

RL95-2 and Ishikawa cells were seeded at 1.2 × 10^6.^ cells in 100-mm dishes for 24 h before treatment. Cells were then exposed to API-59CJ-OMe at 12 or 24 *μ*M for 48–72 h. Protein (500 *μ*g) from cell lysates was immunoprecipitated with either anti-AKT monoclonal antibody or anti-ERK monoclonal antibody. AKT kinase assays and extracellular signal-regulated kinases (ERK) kinase assays were performed using AKT and ERK kinase assay kits (Cell Signaling Tech., Beverly, MA, USA) with glycogen synthase kinase-3 (GSK-3) as a substrate for AKT and Elk-1 as a substrate for ERK. Phospho-specific antibodies to GSK-3 *α*/*β* (Ser 21/9) and Elk-1 (Ser 383) were used for phosphorylated protein detection. At least three independent repetitions were performed for each assay type. An additional assay was performed to verify equal immunoprecipitation of AKT from treated and untreated cells; AKT was immunoprecipitated from cell lysates using the same anti-AKT monoclonal antibody as described above. Cell lysates were incubated and washed as per the kinase assay protocol, but then separated via Western blot prior to the performance of the kinase assay.

### Apoptotic assay

API-59CJ-OMe was synthesised in Dr Shaomeng Wang's laboratory at the University of Michigan. To quantitate the induction of apoptosis by API-59CJ-OMe, cells from all four cell lines were plated at 3 × 10^5^ cells per 6-cm dish 1 day prior to exposure to API-59CJ-OMe at 1.5, 6, 12, or 24 *μ*M. After 72 h of treatment, cells were harvested and fixed with 70% ethanol. Cells were then stained with propidium iodide and analysed for reduction of DNA content (sub-G1 profile) on a FACScan flow cytometer (Becton Dickinson, San Jose, CA, USA). The results given are average values and standard deviations from at least three separate experiments.

## RESULTS

### Expression of PTEN protein and phosphorylated AKT in endometrial cancer cell lines

**Figure 1:**
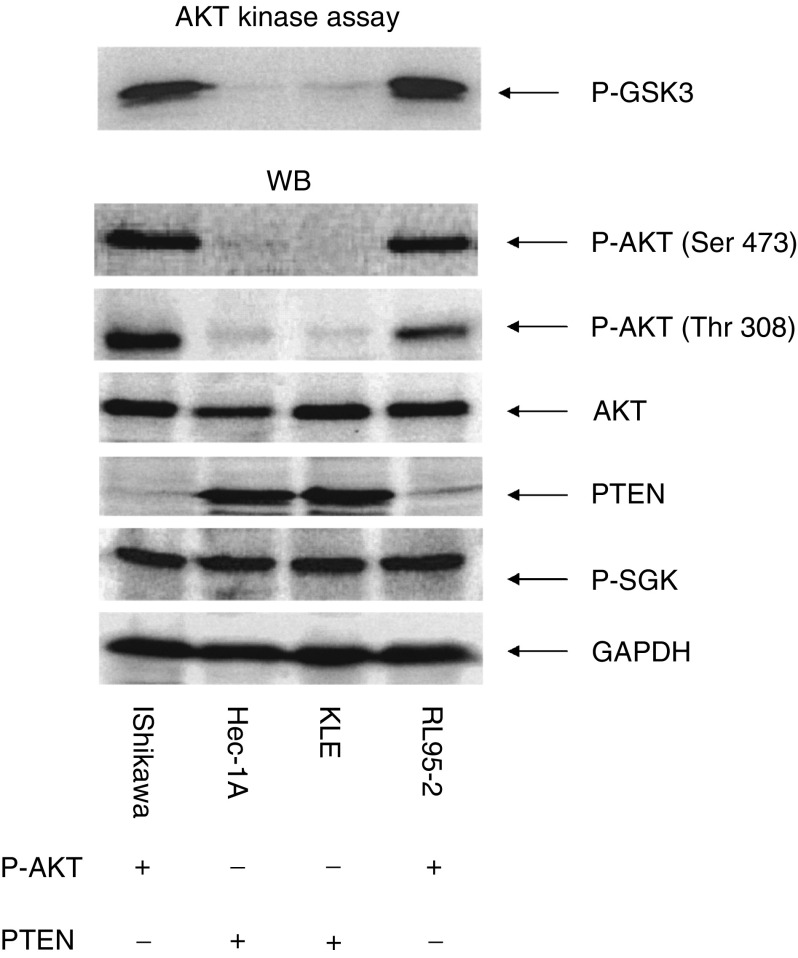
Expression of PTEN and phospho-AKT in endometrial cancer cell lines. Cell lysates were immunoblotted with phosphorylated AKT (Ser 473 or Thr 308), phosphorylated SGK (Ser 78), total AKT, or PTEN antibody. The same lysates were immunoprecipitated with anti-AKT antibody; AKT kinase assay was performed with GSK-3 as a substrate and phospho-specific GSK-3 *α*/*β* (Ser 21/9) for phosphorylated protein detection.

We first examined the expression of PTEN and phosphorylation of AKT in the four human endometrial cancer cell lines, RL95-2, Ishikawa, Hec1A, and KLE. Two of these cell lines, Hec1A and KLE, express a considerable amount of PTEN protein, whereas the other two, Ishikawa and RL95-2, express little or no PTEN protein (Figure 1

). This is consistent with previous reports that Ishikawa and RL95-2 cell lines contain PTEN mutations, while Hec1A and KLE cells express wild-type PTEN (Yaginuma *et al*, 2000). We also found that Ishikawa and RL95-2 cell lines express high levels of phosphorylated AKT and have high AKT kinase activity, whereas Hec1A and KLE cells express little phosphorylated AKT and have undetectable AKT kinase activity (Figure 1). However, phosphorylation of serum- and glucocorticoid-inducible kinase (SGK), an AKT-related serine/threonine kinase, is independent of PTEN status in these cancer cell lines (Figure 1). Therefore, in these four cell lines, there is perfect correlation between loss of PTEN expression and overactivation of AKT kinase.

### Inhibition of AKT kinase activity in human endometrial cancer cell lines

**Figure 2:**
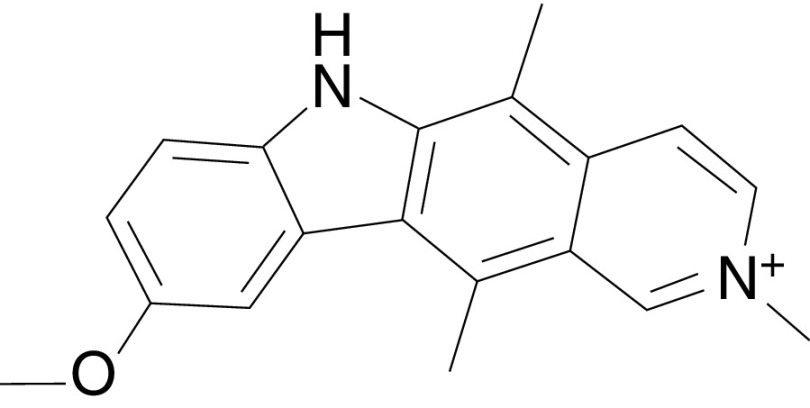
Chemical structure of API-59CJ-OMe, 9-methoxy-2-methylellipticinium acetate.

Through a bioinformatics approach, we have identified a nonpeptide small molecule inhibitor, API-59CJ-OMe, as a potential inhibitor of the AKT pathway (Wang and Yang, manuscript in preparation). The chemical structure of API-59CJ-OMe is shown in Figure 2

**Figure 3:**
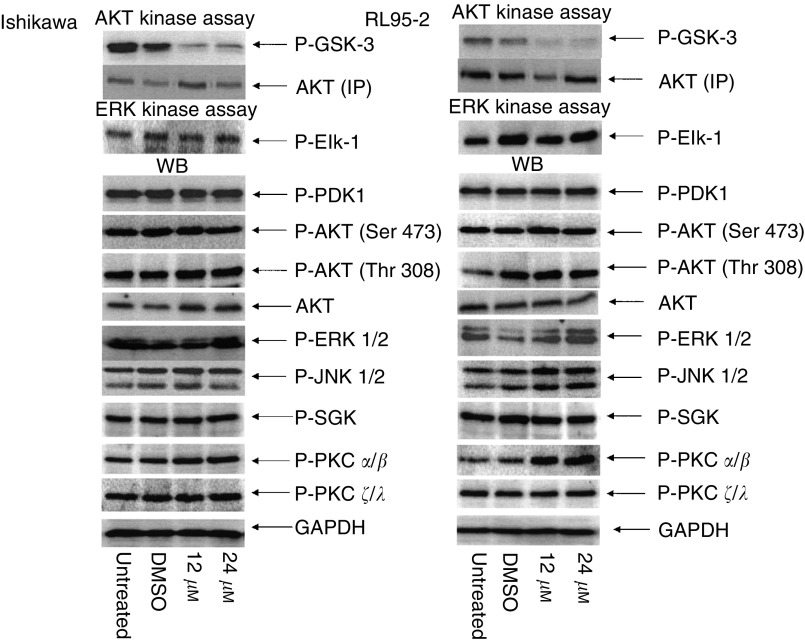
Effect of API-59CJ-OMe on AKT kinase activity and phosphorylation of other kinases. (**A**) After treatment with API-59CJ-OMe, cells were harvested and cell lysates separated by 10% SDS–PAGE. Gels were analysed with phospho-specific antibodies to PDK1 (Ser 241), AKT (Ser 473 or Thr 308), ERK1/2 (Thr 202/Tyr 204), JNK (Thr 183/ Tyr 185), SGK (Ser 78), PKC*ζ*/*λ* (Thr 410/403), and PKC*α*/*β* (Thr 638/641). The same lysates were immunoprecipitated with anti-AKT or anti-ERK antibody, and kinase assays performed with GSK-3 as a substrate for AKT and Elk-1 as a substrate for ERK. AKT (IP) represents the immunoprecipitation control: lysates were immunoprecipitated with mouse monoclonal anti-AKT antibody, separated by SDS–PAGE, and stained with rabbit polyclonal anti-AKT antibody. Phospho-specific antibodies to GSK-3 *α*/*β* (Ser 21/9) and Elk-1 (Ser 383) were used for phosphorylated protein detection.

. We tested whether API-59CJ-OMe can inhibit AKT kinase activity in RL95-2 and Ishikawa cells, the two cell lines with high levels of AKT phosphorylation and kinase activity. Addition of API-59CJ-OMe significantly inhibited AKT kinase activity when GSK-3 is used as a substrate in RL95-2 and Ishikawa cells (Figure 3

). The amount of total AKT immunoprecipitated from cell lysates was not affected by treatment with API-59CJ-OMe. API-59CJ-OMe had no effect on ERK kinase activity. To further demonstrate the selectivity of API-59CJ-OMe, we probed the same cell lysates with antibodies against phosphorylated MAP kinases (ERK1/2 and JNK1/2), phosphorylated AKT, phosphorylated SGK, phosphorylated PKC isoforms, or phosphorylated PDK-1. As shown in Figure 3, API-59CJ-OMe did not inhibit phosphorylation of these proteins. Equal protein loading was demonstrated by blotting the same membranes with GAPDH antibody. API-59CJ-OMe did not affect kinases either upstream of AKT (PDK-1) or in a distinct signal transduction pathway (ERK1/2 and JNK1/2) in endometrial cancer cells. Of note, API-59CJ-OMe did not inhibit phosphorylation of AKT itself at either Serine 473 or Threonine 308. This suggests that API-59CJ-OMe may act at the AKT kinase level and is less likely to act upstream at kinases responsible for either Serine 473 or Threonine 308 phosphorylation.

### Induction of apoptosis in human endometrial cancer cell lines with elevated AKT activity

**Figure 4:**
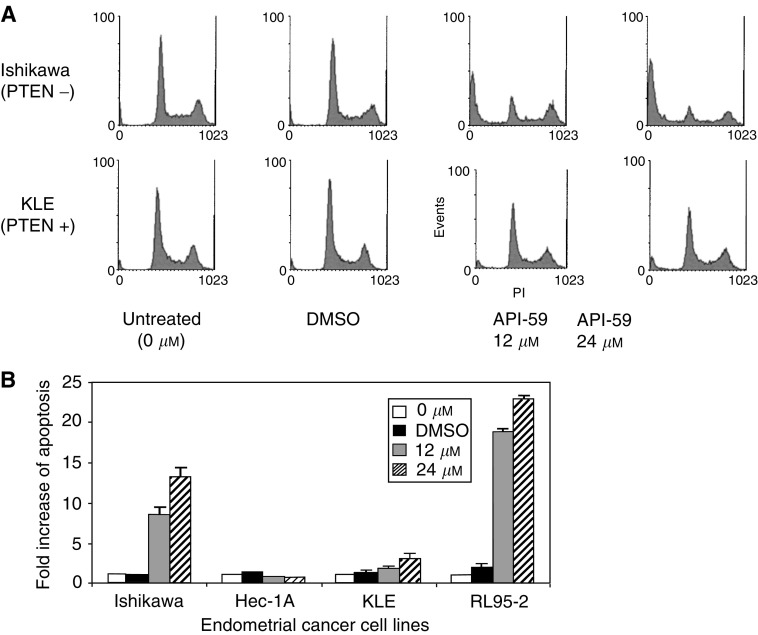
API-59CJ-OMe induces apoptosis of endometrial cancer cells expressing activated AKT. (**A**) Two examples of flow cytometry histograms in Ishikawa and KLE cells; API-59CJ-OMe induced marked apoptosis in Ishikawa and RL95-2 cells, but not in Hec1A or KLE cells. (**B**) Fold increase of apoptosis was calculated as percentage apoptosis in treated cells over percentage apoptosis in untreated cells. Results shown in (**B**) are the average and standard deviation of Log PI from three independent experiments.

We examined whether API-59CJ-OMe could induce apoptosis in RL95-2 and Ishikawa endometrial cancer cell lines. We predicted that inhibition of the AKT survival pathway by API-59CJ-OMe would lead to apoptosis of these cancer cells. As expected, exposure of API-59CJ-OMe at 12 and 24 *μ*M significantly induced apoptosis in both RL95-2 and Ishikawa endometrial cancer cell lines, which express high levels of phosphorylated AKT and AKT kinase activity. API-59CJ-OMe had only minimal effects on Hec1A and KLE, cell lines that express wild-type PTEN and lack AKT kinase activity (Figure 4

). Lower doses (1.5 and 6 *μ*M), when screened using MTT cell viability assays, had minimal effects on cell number, and were significantly less effective at inducing apoptosis in flow cytometry assays (data not shown).

## DISCUSSION

PTEN mutation is a frequent event in human endometrial cancer. Good correlation exists between loss of PTEN expression and high levels of phosphorylated AKT in endometrial cancer cells (Kanamori *et al*, 2001). High AKT kinase activity in cancer cells may provide survival or proliferation signals and promote oncogenesis (Franke *et al*, 1997; Kulik *et al*, 1997). Thus, inhibition of AKT represents an attractive therapy for the treatment of endometrial cancer. Inhibitors that target upstream regulators of AKT such as PI3-K and PDK-1, or potentially target the pleckstrin homology (PH) domain of AKT, have been reported (Hu *et al*, 2000; Ballif *et al*, 2001; Kozikowski *et al*, 2003; Meuillet *et al*, 2003). Introduction of wild-type PTEN into endometrial cancer cells harbouring PTEN mutation can inhibit cell growth (Lilja *et al*, 2001). In this report, we describe a potential small molecule inhibitor of the AKT pathway. Small molecule drugs have several advantages, including good delivery properties, good *in vivo* stability, low probability of immune response, and low cost.

API-59CJ-OMe (or MMEA) belongs to the class of compounds referred to as ellipticines. Ellipticines with a wide variety of substitutions at the 2 and 9 positions have been evaluated as potential antitumour agents, and there is evidence for several different mechanisms of action for this group of drugs. The ellipticines can bind directly to DNA, and the 2, 9 substituted ellipticines may act as DNA ‘threaders’, intercalating into the DNA strands (Harding and Grummitt, 2003). These compounds also stabilise topoisomerase II–DNA complexes, and promote DNA strand breakage. Some studies have also suggested that the ellipticines may act to restore wild-type p53 function in cells with mutant p53, although the results in our lab do not confirm this. Finally, some of the ellipticines (9-hydroxyellipticines) may promote generation of superoxide radicals, leading to DNA strand breakage. Exactly how these described mechanisms relate to the AKT inhibition we see in our system is still unclear, and we are working further to clarify this. Several compounds have been used in Phase I and II human trials for a variety of malignancies. The compound appears to be metabolised by cytochrome P450, and reported toxicities have varied widely across trials, which have mostly involved 9-hydroxy compounds. There is *in vitro* data that suggests that API-59CJ-OMe/MMEA may display some neurotoxicity (Sriram *et al*, 1997). Additional testing to determine the toxicity profiles of API-59-CJOMe will be required before this compound can be considered as a possible human therapeutic. The lack of major effects on the two PTEN-intact cell lines suggests that normal cells may have some innate resistance to the compound. However, this will have to be verified in animal models, as cell culture has only limited capacity to predict *in vivo* toxicity.

Our data demonstrate that API-59CJ-OMe can inhibit AKT kinase activity in cell culture. API-59CJ-OMe does not inhibit ERK kinase, nor did it affect phosphorylation of ERK1/2, JNK1/2, PKC isoforms, SGK, PDK1, or AKT itself. This suggests that API-59CJ-OMe may inhibit the AKT pathway at the AKT level, but not at upstream kinases that phosphorylate AKT at Serine 473 or Threonine 308, in endometrial cancer cells. API-59CJ-OMe induces apoptosis in endometrial cancer cell lines expressing high levels of AKT activity, but has little effect in endometrial cancer cells lacking AKT activity. Further, both Ishikawa and RL95-2 cells harbour endogenous mutant p53 and API-59CJ-OMe did not induce p53 targets, mdm2 (data not shown). This suggests that API-59CJ-OMe does not induce apoptosis through the p53-dependent pathway. AKT is frequently activated in endometrial carcinoma due to PTEN mutation, API-59CJ-OMe has potential clinical applications in endometrial cancer. We plan to further explore inhibition of AKT pathway using this small molecule inhibitor in a nude mouse xenograft model.

In addition to endometrial cancer, PTEN is frequently mutated in brain and prostate cancer (Cairns *et al*, 1997; Li and Sun, 1998). AKT activity is also elevated in many ovarian and breast cancers. In these tumours, AKT activation is mainly due to amplification of the *AKT* oncogene or activation by upstream regulators (Cheng *et al*, 1992; Bellacosa *et al*, 1995; Tashiro *et al*, 1997). It will be of interest to determine whether API-59CJ-OMe can inhibit other cancer types with high AKT kinase activity. Our lab has already shown that API-59CJ-OMe inhibits AKT kinase activity and induces apoptosis in ovarian cancer cells with high AKT activity (Tang and Lin, manuscript submitted). These studies represent the first steps towards developing a small molecule therapy that targets the AKT oncogenic pathway. This approach may prove useful not only against endometrial cancer but against prostate cancer, brain cancer, and other tumours where disruption of PTEN and AKT is common.
